# Upper Alpha Based Neurofeedback Training in Chronic Stroke: Brain Plasticity Processes and Cognitive Effects

**DOI:** 10.1007/s10484-017-9353-5

**Published:** 2017-02-14

**Authors:** Silvia Erika Kober, Daniela Schweiger, Johanna Louise Reichert, Christa Neuper, Guilherme Wood

**Affiliations:** 1 Department of Psychology, University of Graz, Universitaetsplatz 2/III, 8010 Graz, Austria; 2 BioTechMed-Graz, Mozartgasse 12/II, Graz, 8010 Austria; 3 Institute of Neural Engineering, Laboratory of Brain-Computer Interfaces, Graz University of Technology, Stremayrgasse 16, Graz, 8010 Austria

**Keywords:** Cortical reorganization, Neurofeedback, Memory, Stroke recovery

## Abstract

In the present study, we investigated the effects of upper alpha based neurofeedback (NF) training on electrical brain activity and cognitive functions in stroke survivors. Therefore, two single chronic stroke patients with memory deficits (subject A with a bilateral subarachnoid hemorrhage; subject B with an ischemic stroke in the left arteria cerebri media) and a healthy elderly control group (*N* = 24) received up to ten NF training sessions. To evaluate NF training effects, all participants performed multichannel electroencephalogram (EEG) resting measurements and a neuropsychological test battery assessing different cognitive functions before and after NF training. Stroke patients showed improvements in memory functions after successful NF training compared to the pre-assessment. Subject B had a pathological delta (0.5–4 Hz) and upper alpha (10–12 Hz) power maximum over the unaffected hemisphere before NF training. After NF training, he showed a more bilateral and “normalized” topographical distribution of these EEG frequencies. Healthy participants as well as subject A did not show any abnormalities in EEG topography before the start of NF training. Consequently, no changes in the topographical distribution of EEG activity were observed in these participants when comparing the pre- and post-assessment. Hence, our results show that upper alpha based NF training had on the one hand positive effects on memory functions, and on the other hand led to cortical “normalization” in a stroke patient with pathological brain activation patterns, which underlines the potential usefulness of NF as neurological rehabilitation tool.

## Background

Following stroke, changes in electrical brain activity as well as cognitive impairment are often evident (Melkas et al. 2014; Jordan 2004; Kaplan and Rossetti 2011; Finnigan and van Putten 2013; Niedermeyer 2005). In this context, EEG based neurofeedback (NF) might be a useful rehabilitation tool. There is evidence that NF training can lead to changes in electrical brain activity which goes along with cognitive improvements (Kober et al. 2015a, b; Reichert et al. 2016; Kropotov 2009; Gruzelier 2014). Using NF, participants can learn to voluntarily modulate their electrical brain activity. Specific parameters of the EEG, such as power values in specific frequency bands, can be extracted and analyzed in real-time and fed back to the participants via auditory and/or visual feedback. Hence, with the method of NF, the electrical activity of the brain is modulated directly and, therefore, the cortical substrates of cognitive functions. This direct access to neural activity by means of NF may alter or accelerate functional reorganization in the brain following stroke. NF might speed up functional recovery or even enable functional recovery that otherwise would not have occurred (Nelson 2007). Therefore, the aim of the present study was to evaluate the effects of EEG based NF training on brain plasticity processes and cognitive functions in stroke survivors.

It has been demonstrated that the electroencephalogram (EEG) is a highly sensitive measure to detect cerebral ischemic or hemorrhagic stroke. Stroke patients show EEG abnormalities compared to healthy people, which change over the course of the disease. The quantitative EEG during the acute and sub-acute state has a high prognostic value concerning the outcome from stroke (Finnigan et al. 2007; Finnigan and van Putten 2013; Tecchio et al. 2007; Sheorajpanday et al. 2011). In this context, slow wave EEG activity in the delta range (0.5–4 Hz) as well as faster oscillatory activity in the alpha range (8–12 Hz) turned out to play an essential role (Niedermeyer 2005). Delta power was found to be negatively correlated with regional cerebral blood flow (rCBF) while alpha power showed a relatively strong positive correlation with rCBF (Tolonen and Sulg 1981; Finnigan and van Putten 2013). In stroke patients with unilateral cerebral infarction delta activity is typically most pronounced over the affected hemisphere in the acute state (Finnigan and van Putten 2013; Jordan 2004; Tecchio et al. 2006). Across a few hours during the acute stroke period, the scalp topography of delta activity shifts from a maximum over the affected hemisphere to a maximum over the healthy, unaffected hemisphere. This interhemispheric shift of scalp delta power maxima is associated with worsening of cerebral pathophysiology and clinical state in stroke patients (Finnigan et al. 2008; Tecchio et al. 2007; Zappasodi et al. 2007; Niedermeyer 2005; Rossini et al. 2003). There is evidence that pathological asymmetry in EEG delta power decreases after thrombolytic therapy, which in turn leads to improvements in clinical symptoms (Finnigan et al. 2006; de Vos et al. 2008). Alpha amplitude attenuation is also generally indicative for cortical injury (Finnigan and van Putten 2013; Finnigan et al. 2007; Klimesch 1999). Alpha power in the acute state is negatively related to the severity of stroke symptoms in patients with unilateral lesions in the arteria cerebri media (ACM) (Finnigan et al. 2007). In stroke patients with acute subarachnoid hemorrhage (SAH), EEG delta activity is increased and alpha activity is reduced, too (Vespa et al. 1997; Claassen et al. 2004; Niedermeyer 2005; Labar et al. 1991). Some EEG studies also investigated changes in EEG activity in the post-acute and chronic stage (Mattia et al. 2003). These studies showed that the greatest improvement in EEG activity occurred during the first 3 months after stroke (Giaquinto et al. 1994; de Weerd et al. 1988; Jonkman et al. 1984). Stroke patients with a unilateral insult in the ACM showed decreases in delta power and increases in alpha power levels over the injured hemisphere during this time period. Alpha power levels also increased over the healthy hemisphere. An overall increase in alpha power was also partially associated with improvements in motor functions and activities in daily living (Giaquinto et al. 1994). Furthermore, delta and alpha power became more symmetrically distributed over both hemispheres with clinical recovery, which might be an indicator of “normalization” of electrical brain activity (Giaquinto et al. 1994; Tecchio et al. 2006).

In the present investigation, we evaluated whether EEG based NF training can be used as therapeutic tool to evoke changes in electrical brain activation patterns in chronic stroke patients, which may be accompanied by cognitive improvements. In NF training paradigms, participants can learn to voluntarily increase or decrease the amplitude of specific EEG frequencies. There is some empirical evidence that voluntary modulation of EEG amplitudes determines other aspects of electrical brain activity in healthy people, which are responsible for improved cognitive performance (Egner et al. 2004; Egner and Gruzelier 2004; Kropotov et al. 2005; Kober et al. 2015a; Reichert et al. 2016). A few single-case studies in stroke patients reported heterogeneous results. Some found positive effects of NF training on cognitive functions as well as a EEG normalization after NF training (Rozelle and Budzynski 1995; Bearden et al. 2003; Laibow et al. 2002; Putman 2002; Hofer et al. 2014), others could not find any significant effects (Doppelmayr et al. 2007). However, the generalizability of these prior findings is limited due to the incomplete description of training-specific EEG signal changes as well as the absence of control groups. The majority of NF training studies examined the effects of NF only on the behavioral level (see Gruzelier 2014 for a review). Generally, successful modulation of EEG band power is associated with cognitive and behavioral improvements (Kropotov 2009; Gruzelier 2014; Kober et al. 2015a, b; Hofer et al. 2014; Reichert et al. 2016). For instance, voluntary up-regulation of the upper alpha frequency band (UA, about 10–12 Hz) generally leads to improvements in working memory (WM) and short-term memory performance (Escolano et al. 2011, 2012, 2013, 2014; Angelakis et al. 2007; Nan et al. 2012). It is assumed that alpha activity inhibits unnecessary or conflicting processes to the task being performed, thus facilitating attention and memory by actively suppressing distracting stimuli (Klimesch et al. 2007). Beside voluntary modulation of the magnitude of EEG amplitudes, NF can be also used to change the topographical distribution of EEG activity. For instance, NF is used to tread depressive symptoms by changing hemispheric asymmetry in alpha band power (8–12 Hz) in prefrontal brain areas (Kropotov 2009).

Summing up, we aimed at investigated the effects of NF training on (i) electrical brain activity, such as power in different EEG frequencies and the topographical distribution of EEG activity, and (ii) cognitive functions in chronic stroke patients. Therefore, we present two cases, a stroke patient with a unilateral middle cerebral artery (ACM) stroke and a stroke patient with a bilateral subarachnoid hemorrhage (SAH). We used an UA based NF training, since the two chronic stroke patients showed deficits in memory functions prior to the NF training. Based on the literature, UA based NF training should have specific positive effects on memory functions (Escolano et al. 2011, 2012, 2013, 2014; Angelakis et al. 2007; Nan et al. 2012). We compared the results of the two stroke patients to the results of a healthy, elderly control group. We expected that pathological EEG patterns in stroke patients would change due to UA based NF training, which should be associated with cognitive recovery.

## Methods

### Participants

**Fig. 1:**
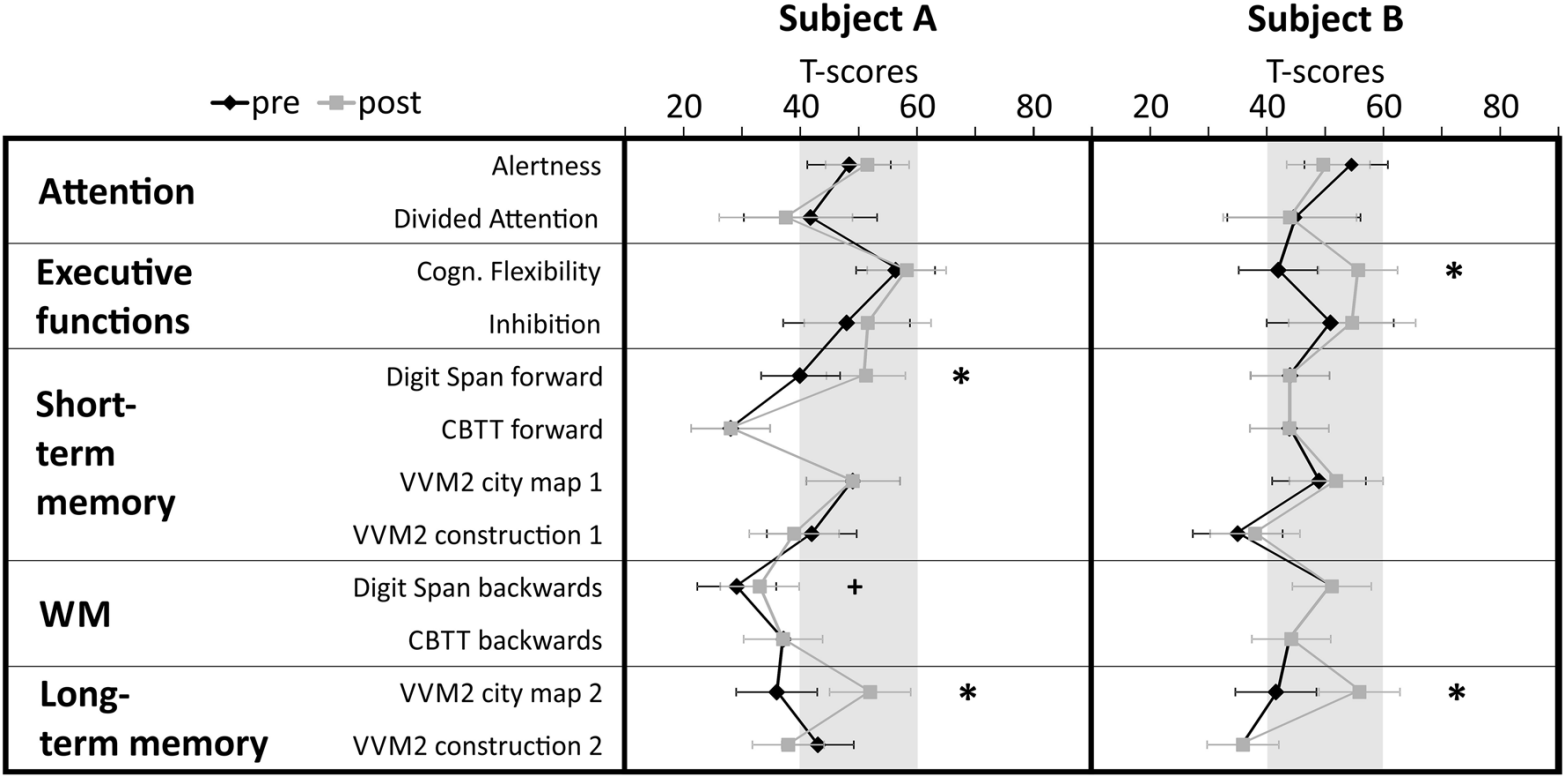
Test performance is expressed in T-scores with population mean *M* = 50 and standard deviation *SD* = 10. Single subject test scores and confidence intervals for measurements of attention, executive functions, short- and long-term memory, and working memory (WM) performed during the pre- and post-assessment are depicted separately for stroke patient A and B. Significant differences between pre- and post-test (critical difference analysis on the group level, Huber 1973) are marked with asterisks (*significant, ^+^marginally significant). *CBTT* corsi block tapping test, VVM visual and verbal memory test

Two stroke patients in a chronic state participated in the NF training. Subject A was a 72-year-old right-handed man who suffered a non-traumatic subarachnoid hemorrhage (time since onset 27 months) at parieto-temporal regions and a concomitant non-traumatic intracerebral hemorrhage resulting in bilateral lesions in occipital–parietal and frontal brain regions. He had no motor deficits. The neuropsychological test-battery assessed before the start of the NF training revealed cognitive deficits in different memory functions (T-scores <40) (Fig. 1). Spatial, personal and temporal orientation of subject A were intact. Subject B was a 73-year-old right-handed man who suffered a mono-hemispheric ischemic stroke (time since onset 70 months). The cerebral infarction was due to a thrombosis of the left arteria cerebri media (ACM). The patient had no motor deficits or severe cognitive deficits. In the neuropsychological pre-assessment he showed some deficits in short- and long-term memory tasks (T-scores < 40) (Fig. 1). His spatial, personal and temporal orientation were also intact.

Patient exclusion criteria were a visual hemi-neglect, dementia (defined as a score of <24 in the mini-mental state examination, MMSE Kessler et al. 1990), psychiatric disorders such as depression or anxiety, other concomitant neurological disorders (e.g. Parkinson disease; visual-reflex epilepsy), aphasia, drug therapies that interfere with the vigilance state, or insufficiently motivation and cooperation. Patients and healthy elderly controls had normal or corrected-to-normal vision and hearing.

A neurologically healthy control group (CG) (*N* = 24, 33% male, mean age 63 years) was recruited as well. All healthy participants disavowed any current or previous psychiatric or neurological disorders. They received an expense allowance of 7 Euro per hour. The healthy CG showed no deficits in any test parameter of the neuropsychological assessment (all T-scores between 40 and 60).

All participants gave written informed consent to participate prior to their inclusion in the study. We have also obtained consent to publish from the participant and to report individual patient data. The study was approved by the local ethics committee of the University of Graz (Reference No. GZ. 39/22/63 ex 2012/13 and GZ. 39/11/63 ex 2013/14) and was in line with the code of ethics of the World Medical Association, Declaration of Helsinki.

### Pre-post-assessment

#### Neuropsychological Assessment of Cognitive Functions

To evaluate possible effects of NF training on cognitive functions, before and after the NF training all participants performed standardized neuropsychological tests to assess attention, executive functions, short- and long-term memory as well as working memory functions.

To assess the state of active attention, the subtest *Alertness* of the Test of Attentional Performance (TAP) test battery (Zimmermann and Fimm 2007–2012) was administered. Divided attention was assessed with the subtest *Divided Attention* of the TAP test battery (Zimmermann and Fimm 2007–2012) and inhibitory processes were assessed with the subtest *Go*/*NoGo* of the TAP test battery (Zimmermann and Fimm 2007–2012). To investigate cognitive flexibility we used the subtest *Flexibility* of the TAP (Zimmermann and Fimm 2007–2012). Verbal long-term memory was measured by using the Visual and Verbal Memory Test (VVM2) subscale *Construction 2* and non-verbal/visual–spatial long-term memory was measured with the VVM2 subscale *City map 2* (Schelling and Schächtele 2001). Verbal short-term memory was assessed with the VVM2 subscale *Construction 1* (Schelling and Schächtele 2001) and with the forward task of the *Digit Span test* of the Wechsler Memory Scale (WMS-R; Härting and Wechsler 2000). Non-verbal or visual–spatial short-term memory was assessed with the VVM2 subscale *City map 1* (Schelling and Schächtele 2001) and the forward task of the *Corsi Block Tapping Test* (CBTT; Härting and Wechsler 2000). Additionally, the backwards tasks of the *Digit Span test* and the *CBTT* were used to assess working memory performance (Härting and Wechsler 2000). Parallel forms of the memory tests were used to avoid learning effects.

#### EEG Resting Measurements

Before and after the NF training, all participants performed EEG resting measurements with closed and open eyes á 3 min. For these multi-channel EEG recordings, a BrainAmp Standard amplifier (Brain Products GmbH, Munich, Germany) was used. EEG was recorded by Ag/AgCl electrodes from 60 electrode positions according to the extended 10–20 electrode placement system against a linked mastoid reference, the ground was placed at FPz. Vertical and horizontal EOG was recorded with three electrodes in total, two were placed on the outer canthi of the eyes and one was placed superior to the nasion. Electrode impedances were kept below 5 kOhms for the EEG recording and below 10 kOhms for the EOG recording. EEG signals were digitized at 500 Hz and filtered with a 0.01 Hz high-pass and a 100 Hz low-pass.

### Upper Alpha Neurofeedback Training

For the upper alpha NF training, EEG signal was recorded using a 10-channel amplifier (NeXus-10 MKII, Mind Media BV) with a sampling frequency of 256 Hz, the ground was located at the right mastoid, the reference was placed at the left mastoid. The feedback electrode was placed over Pz. One EOG channel was recorded at the left eye. The NF paradigm was generated by using the software BioTrace+ (Mind Media BV, Kober et al. 2013). Up to ten NF training sessions were carried out on different days 3–5 times per week. Each session lasted approximately 45 min and consisted of a baseline run and six feedback runs á 3 min each. During the feedback runs, participants were rewarded by getting points when they increased their upper alpha power above an individually predefined threshold (mean power of baseline run and previous runs), while keeping other frequencies in the theta and beta range, which reflect eye blinks/movements and muscle activity respectively, below a certain threshold (mean power of baseline run + 1 SD) (Weber et al. 2011; Doppelmayr and Weber 2011). Visual feedback was provided by vertically moving bars depicting the power values in the feedback frequency bands. The upper alpha frequency range, which was used as feedback frequency during NF training, was defined individually for each single participant. Therefore, the EEG resting measurements of the pre-assessment were used to calculate the EEG power spectrum for each participant. EEG power spectra were calculated using Fast Fourier Transformation (FFT). FFT was computed for the segmented resting measurements (segment length 1 s) with maximum resolution of ~0.98 Hz. Furthermore, a 10% Hanning window was applied including a variance correction to preserve overall power. Afterwards, peak detection in the Alpha frequency range was performed to identify the individual alpha frequency (IAF). The upper and lower alpha band were defined in the following way (Klimesch 1999): lower alpha = (IAF −2 Hz) to IAF; upper alpha = IAF to (IAF + 2 Hz). Both stroke patients showed a clear alpha peak at 10 Hz during the EEG resting measurements of the pre-assessment (IAF = 10 Hz). The CG showed a mean IAF of 9.25 Hz (*SE* = 0.20). No significant changes in IAF values due to NF training could be observed.

For statistical analysis, UA power values of the NF training sessions were z-transformed to ensure comparability across sessions and subjects. To analyze more closely the time course of UA power over the training runs averaged over all sessions, we conducted linear regression analyses (predictor variable = run; dependent variable = UA power). As during successful NF, a within-session increase of the feedback frequency power is expected, the slope of the regression line was used as an indicator of NF performance (see Escolano et al. 2012; Witte et al. 2013; Zoefel et al. 2011; Kober et al. 2015a).

### EEG Data Preprocessing and Analysis

Data analysis of all EEG recordings (EEG resting measurements during pre- and post-assessment and NF training data) was performed offline using the Brain Vision Analyzer software (version 2.01, Brain Products GmbH, Munich, Germany). Artefacts (e.g. muscle activity) were rejected by means of a semi-automatic artefact rejection (criteria for rejection: > 50.00 µV voltage step per sampling point, absolute voltage value > ± 120.00 µV). Ocular artefacts (eye blinks) were automatically corrected using the algorithm developed by Gratton, Coles and Donchin (1983) (Gratton et al. 1983). All epochs with artefacts were excluded from the EEG analysis. To analyze the feedback training data, absolute values of upper alpha (IAF to (IAF + 2 Hz)) power were calculated and averaged separately for each 3-min run of each sessions using the Brain Vision Analyzer’s built-in method of complex demodulation (method to calculate power, Brain Products GmbH 2009). The complex demodulation is based on the complex (analytical) signal of a time series, where all frequencies except the one of interest are filtered out (Draganova and Popivanov 1999). To analyze the EEG resting measurements, absolute values of delta 0.5-4 Hz, theta 4–8 Hz, low alpha 8–10 Hz, upper alpha 10–12 Hz, low beta 12–15 Hz, mid beta 15–21 Hz, high beta 21–35 Hz, and gamma 35–45 Hz power in a fixed range were calculated and averaged separately for the eyes-open and eyes-closed condition using the Brain Vision Analyzer’s built-in method of complex demodulation. Note that no NF training effects were found in theta, low alpha, beta, and gamma bands for the resting measurements. Therefore, only the analysis of EEG power in the delta and upper alpha band will be reported.

### Description of Statistical Analysis

In order to analyse the NF training performance, we determined the time course of UA power averaged over the NF training sessions across the six feedback runs using linear regression analysis (see 2.3). In addition, one-sample *t* tests against 0 were calculated for the CG to verify the consistency of the learning effects. The slopes of the regression line of the single stroke patients were statistically compared to the slopes of the CG by applying single-case analysis methods (Crawford and Garthwaite 2002). These methods enable the assessment of the probability that test scores and test score discrepancies of a single patient and a modest-sized control sample belong to the same distribution (Crawford and Garthwaite 2002).

To analyze the results of the neuropsychological assessment of cognitive functions, T-scores of the single neuropsychological test parameters were used. To investigate the effects of NF on cognitive functions, we conducted intra-individual comparisons between cognitive performance assessed during pre- and post-assessment by using critical difference analysis (Huber 1973). To identify significant improvement or decline for each participant, the critical difference of the relevant test parameter was compared with the test score difference obtained during the post-assessment minus the pre-assessment. To establish the critical difference for a pair of test scores, a correction for measurement error based on the test–retest reliability of the test is performed. The difference between pre- and post-assessment shown by the single participants is considered significant when it is larger than the critical difference, which can be detected by each test (error probability α < 5%) and only occurs in the population with a probability lower than α < 10%. Differences in T-scores between pre- and post-assessment for each cognitive test parameter were compared with critical differences on the single subject level as well as on the group level.

**Fig. 2:**
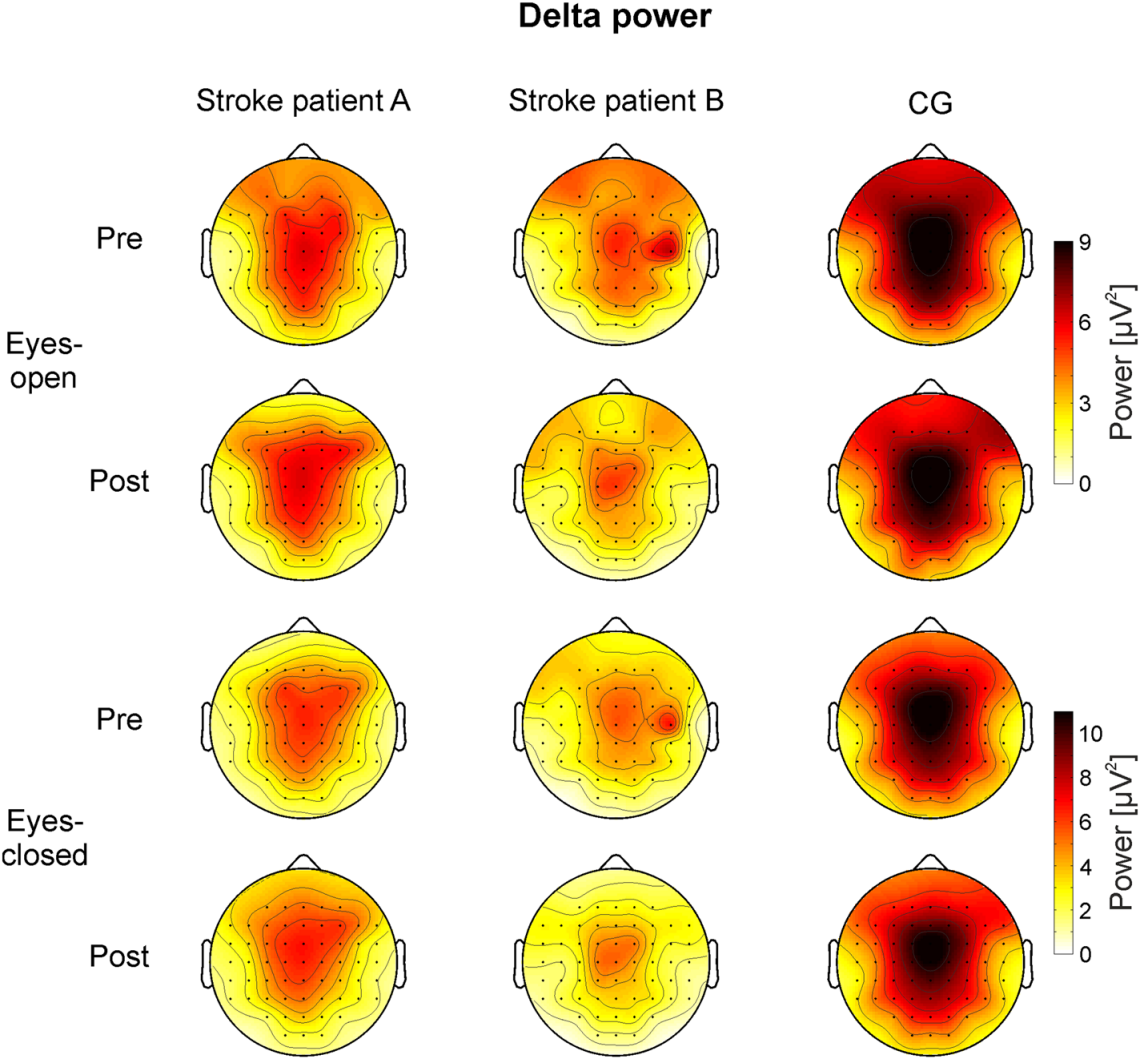
Topographical maps of delta power during the eyes-open (*upper two rows*) and eyes-closed (*lower two rows*) conditions assessed during the pre- and post-test, presented separately for the two single stroke patients and the healthy CG

**Fig. 3:**
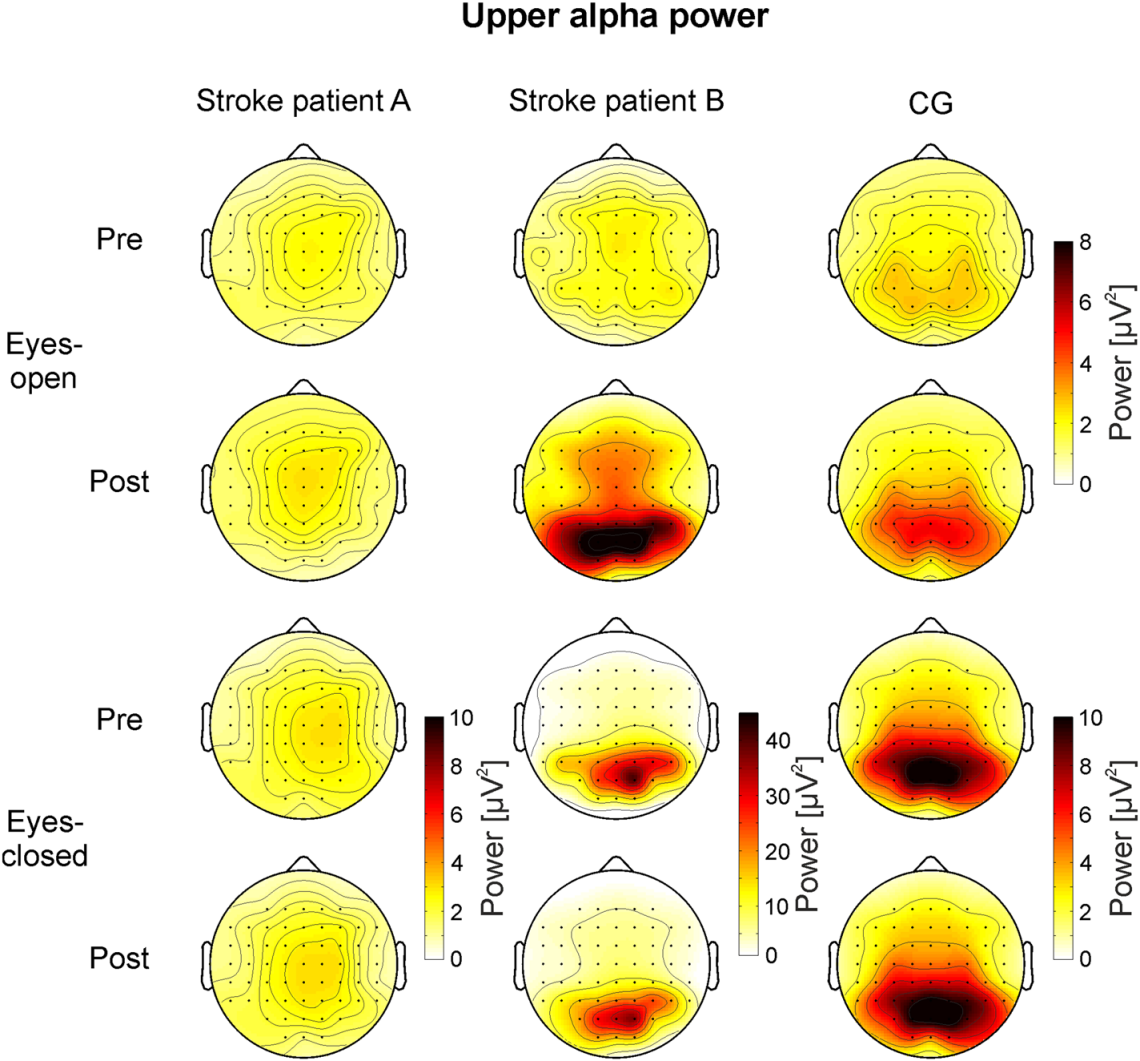
Topographical maps of upper alpha power during the eyes-open (*upper two rows*) and eyes-closed (*lower two rows*) conditions assessed during the pre- and post-test, presented separately for the two single stroke patients and the healthy CG. Note the different scaling of EEG upper alpha power between subjects in the eyes-closed condition

To analyze the EEG activity of the CG during rest, 2 × 3 × 3 univariate repeated-measures analyses of variance (ANOVA) with the within-subjects factors TIME (pre- vs. post-assessment), ACP (anterior vs. central vs. posterior electrode positions) and LATERALITY (left vs. midline vs. right electrode positions) were calculated separately for delta and upper alpha power and for the eyes-closed and eyes-open resting condition. In the CG, delta power was largest over FCz (see Fig. 2). Therefore, the following electrodes were chosen for statistical analysis of the topographical distribution of delta power in the CG: FC5, FCz, FC6, C5, Cz, C6, CP5, CPz, and CP6. Upper alpha power was most pronounced at parieto–occipital sites in healthy controls (see Fig. 3). For the statistical analysis of the topographical distribution of upper alpha power in the CG we chose the following electrodes: CP3, CPz, CP4, P3, Pz, P4, PO3, POz, and PO4.

For the single stroke patients, we calculated difference values (laterality values) in delta and upper alpha power between electrodes of the left and right hemisphere. These laterality values of the single stroke patients were statistically compared to the laterality values of the CG by applying single-case analysis methods (Crawford and Garthwaite 2002). Difference values in EEG power were also calculated between the pre- and post-test to reveal any alterations in absolute EEG power due to NF training.

For all analyses, the probability of a Type I error was maintained at 0.05. For the ANOVA’s, Mauchly’s tests of sphericity were carried out on the repeated-measures variables, and where violated, Greenhous–Geisser correction was applied. For post-hoc analyses, Bonferroni corrections for multiple comparisons were applied.

## Results

### NF performance

Both stroke patients as well as the CG showed a linear increase in UA power across the training runs within the NF training sessions as indicated by positive regression slopes (regression slope: subject A 0.19; subject B 0.18; CG 0.05). The regression model was significant for subject A and the CG (*p* < 0.05), while the regression model was by trend significant for subject B (*p* = 0.08). For the CG, a one-sample t-test revealed that the linear regression slopes of healthy controls were significantly larger than zero (*t* (23) = 2.15, *p* < 0.05). When analyzing the time course of UA power over the training runs separately for each participant of the CG, 15 out of 24 participants (i.e. 62.5%) showed a positive gradient of the learning curve. One of the remaining participants showed a flat learning curve gradient and eight a negative gradient. A single-case analysis (Crawford and Garthwaite 2004) indicated that the regression slope of subject A (*t* (23) = 1.15, *p* = 0.26, effect size for difference between case and controls = 1.18) and subject B (*t* (23) = 1.04, *p* = 0.31, effect size for difference between case and controls = 1.06) did not differ significantly from the healthy participants’ slopes, indicating that the ability of stroke patients to alter UA power was not different from that of healthy controls. The increase in UA power observed in stroke patients and controls across training runs indicated successful NF training.

### Effects on Cognitive Functions

After the NF training, subject A showed significant improvements in short-term memory and long-term memory tasks (Digit Span forward task, VVM2 subscale *City map 2*). His working memory performance also slightly increased although this pre-post difference did not exceed the critical difference value (Fig. 1).

Subject B also showed performance improvements after NF training compared to the pre-assessment. The strongest increase in performance could be observed in a long-term memory task, the subscale *City map 2* of the VVM2. Furthermore, subject B showed an improved performance in the subtest *Flexibility* of the TAP after NF compared to the pre-assessment (Fig. 1).

The CG showed no significant changes in cognitive functions when comparing the pre- and post-assessment.

### Effects on EEG Activity During Rest

#### Delta Power

Figure 2 illustrates the topographical distribution of delta power during the pre- and post-measurements separately for the eyes-open and eyes-closed condition for the two single stroke patients and the healthy CG. In the CG, delta power showed a maximum over fronto-central midline electrodes. The 2 × 3 × 3 ANOVA for the eyes-open condition revealed a significant main effect ACP (*F* (2,46) = 17.87, *p* < 0.01, *η* ^*2*^ = 0.44). Delta power was higher at fronto–central sites (FC: *M* = 6.72 µV^2^, *SE* = 0.48) than at central (C: *M* = 6.23 µV^2^, *SE* = 0.46) and centro-parietal sites (CP: *M* = 6.01 µV^2^, *SE* = 0.44). The main effect LATERALITY was significant, too (*F* (2,46) = 96.53, *p* < 0.01, *η* ^*2*^ = 0.81). Posttests revealed that delta power was highest at midline electrode positions (*M* = 9.49 µV^2^, *SE* = 0.73) than over the left (*M* = 4.66 µV^2^, *SE* = 0.34) or right (*M* = 4.81 µV^2^, *SE* = 0.35) hemisphere. Hence, the CG showed a bilateral distribution of delta power. Furthermore, the interaction effect ACP*LATERALITY was significant (*F*(4,92) = 5.97, *p* < 0.01, *η* ^*2*^=0.21). Posttests indicated a lower delta power over CPz than over FCz and Cz.

For the eyes-closed condition, the ANOVA revealed comparable results than for the eyes-open condition. The main effect ACP (*F* (2,46) = 13.59, *p* < 0.01, *η* ^*2*^=0.37) was significant. Delta power was higher at fronto-central sites (FC: *M* = 7.74 µV^2^, *SE* = 0.73) than at central (C: *M* = 7.08 µV^2^, *SE* = 0.67) and centro-parietal sites (CP: *M* = 6.74 µV^2^, *SE* = 0.61). The main effect LATERALITY was significant, too (*F*(2,46) = 57.15, *p* < 0.01, *η* ^*2*^ = 0.71). Posttests revealed that delta power was highest at midline electrode positions (*M* = 10.97 µV^2^, *SE* = 1.13) than over the left (*M* = 5.19 µV^2^, *SE* = 0.44) or right (*M* = 5.40 µV^2^, *SE* = 0.47) hemisphere. The interaction effect ACP*LATERALITY was also significant (*F* (4,92) = 7.58, *p* < 0.01, *η* ^*2*^ = 0.25). Posttests indicated a lower delta power over CPz than over FCz and Cz.

As illustrated in Fig. 2, subject B showed an increased delta power over the right hemisphere during the pre-assessment. During the post-assessment, subject B showed a central maximum of delta power, which was comparable to the bilateral distribution of delta power in the CG. In contrast, subject A showed no such shift in delta power when comparing the pre- and post-assessment. Delta power was maximal over central electrode positions in subject A during both time points. To test whether these hemispheric differences in subject B were statistically significant, we performed single-case analysis (Crawford and Garthwaite 2002). Therefore, we calculated the difference value of delta power over the right (C6) minus the left (C5) hemisphere (laterality values). The mean laterality values in delta power between the right and left hemisphere are summarized in Table 1. One-sample *t* tests revealed that the laterality values of the CG did not differ significantly from zero (all *p* > 0.05). These results indicate that the CG showed no laterality effects in delta power. During the pre-assessment, subject B showed a significant larger laterality value than the CG during the eyes-open and eyes-closed condition. During the post-assessment, no significant differences in laterality values between subject B and the CG could be observed. Subject A did not differ in his laterality values from the CG during all conditions.

**Table 1:** Laterality values in delta power between the right and left hemisphere (difference value: C6-C5), presented separately for the two single stroke patients and the CG

	Difference in delta power between right and left hemisphere (C6-C5)		
	CG (Mean and *SE*)	Subject A	Subject B
Pre assessment—eyes-open condition	0.17 (0.13)	0.85	2.21*
Post assessment—eyes-open condition	0.15 (0.12)	0.37	0.06
Pre assessment—eyes-closed condition	0.12 (0.16)	0.86	2.43*
Post assessment—eyes-closed condition	0.35 (0.12)	0.19	0.23

Significant differences between the CG and single subjects (single-case analysis, Crawford and Garthwaite 2002) are marked with asterisks (*p < 0.05)

#### Upper Alpha Power

Figure 3 illustrates the topographical distribution of UA power during the different conditions for the two single stroke patients and the healthy CG. In the CG, UA power showed a maximum over parieto–occipital electrodes. The 2 × 3 × 3 ANOVA for the eyes-open condition of the CG revealed no significant results. After NF training, UA power values were numerically higher (Post: *M* = 4.53 µV^2^, *SE* = 1.42) compared to the pre-assessment (Pre: *M* = 2.62 µV^2^, *SE* = 0.39), although the main effect of TIME (*F* (1,23) = 2.16, *p* = 0.15, *η* ^*2*^ = 0.09) did not reach significance.

For the eyes-closed condition, the ANOVA revealed a significant main effect ACP (*F* (2,46) = 9.47, *p* < 0.01, *η* ^*2*^ = 0.29). UA power was higher at parietal (P: *M* = 9.14 µV^2^, *SE* = 2.05) and parieto–occipital (PO: *M* = 10.34 µV^2^, *SE* = 2.17) sites compared to centro-parietal sites (CP: *M* = 6.65 µV^2^, *SE* = 1.32). No hemispheric differences could be observed in the CG.

In the eyes-open condition, subject B showed no prominent changes in the topographical distribution of UA power when comparing the pre- and post-assessment. However, after NF training UA power was stronger over parieto-occipital sites compared to the pre-test. For statistical comparison, we compared the post minus pre differences in UA power of subject B with the same difference values of the CG using single-case analysis (Crawford and Garthwaite 2002). Subject B showed a statistically stronger increase in UA power between pre- and post-test compared to controls over parieto-occipital electrode positions (Table 2). Subject A showed no changes in absolute UA power values when comparing the pre- and post-assessment.

**Table 2:** Difference values in upper alpha power during the eyes-open condition between the post- and pre-assessment for parietal and parieto–occipital electrode positions, presented separately for the two single stroke patients and the CG

	Difference in upper alpha power between post- and pre-assessment		
	CG (Mean and *SE*)	Subject A	Subject B
P3	1.92 (0.43)	0.21	4.22
Pz	2.67 (0.30)	0.23	4.64
P4	1.98 (0.43)	0.06	5.24
PO3	2.16 (0.64)	0.10	8.19^+^
POz	1.95 (0.49)	0.10	7.78*
PO4	2.37 (0.67)	0.02	8.30^+^

Significant differences between the CG and single subjects (single-case analysis, Crawford and Garthwaite 2002) are marked with asterisks (*p < 0.05; ^+^ *p* < 0.10)

In the eyes-closed condition, UA power of subject B was more pronounced over the right compared to the left hemisphere during the pre-assessment, while during the post-assessment the topographical distribution became more bilateral (Fig. 3). Subject A showed no prominent UA maximum, neither during the eyes-open nor during the eyes-closed condition. The CG showed a bilateral distribution of UA power during all conditions. Single-case analysis (Crawford and Garthwaite 2002) revealed a stronger hemispheric difference in subject B compared to the CG at different posterior electrode positions during the pre-assessment. During the post-assessment, these hemispheric differences in subject B did not differ significantly from the laterality values of healthy controls. The mean laterality values in UA power between the right and left hemisphere are summarized in Table 3. One-sample *t* tests revealed that the laterality values of the CG did not differ significantly from zero (all *p* > 0.05).

**Table 3:** Laterality values in upper alpha power between right and left hemisphere (difference values: CP4−CP3, P4−P3, PO4−PO3), presented separately for the two single stroke patients and the CG

	CG (Mean and *SE*)	Subject A	Subject B
Difference in upper alpha power between right and left hemisphere (CP4-CP3)			
Pre assessment—eyes-closed condition	−0.41 (0.49)	0.76	6.66*
Post assessment—eyes-closed condition	−0.03 (0.57)	0.44	4.31
Difference in upper alpha power between right and left hemisphere (P4-P3)			
Pre assessment—eyes-closed condition	0.24 (0.59)	0.66	12.93*
Post assessment—eyes-closed condition	1.22 (0.78)	0.37	6.87
Difference in upper alpha power between right and left hemisphere (PO4-PO3)			
Pre assessment—eyes-closed condition	0.38 (0.71)	0.40	14.96*
Post assessment—eyes-closed condition	0.95 (0.78)	0.31	7.64

Significant differences between the CG and single subjects (single-case analysis, Crawford and Garthwaite 2002) are marked with asterisks (*p < 0.05)

## Discussion

Here we show that EEG based NF training led to cortical reorganization in a stroke patient with pathological EEG activation patterns. Furthermore, UA based NF training led to improvements in memory functions in stroke survivors that showed memory deficits prior to the training. In the following, these results are discussed in more detail.

### NF Performance

In a first step, we could show that the two stroke patients were able to voluntarily increase their upper alpha power through NF training. NF performance of stroke patients and healthy controls was comparable. The CG as well as the stroke patients showed a linear increase in the trained frequency band over the feedback runs, indicating successful NF training (Kober et al. 2015a). These results support previous single-case studies showing that stroke patients are able to control their electrical brain activity during NF training (Bearden et al. 2003; Cannon et al. 2010; Reichert et al. 2016) and extend the evidence by showing that the stroke patients were equally able to up-regulate upper alpha activity as age-matched healthy controls. In line with previous findings in healthy participants, about 1/3 of healthy controls were not able to modulate their EEG activity (Allison and Neuper 2010). The inability to control the own brain activity may be attributed to different factors such as differences in brain structure (Allison and Neuper 2010), inter-individual differences in neurophysiological and psychological factors, or cognitive strategies (Kober et al. 2013; Witte et al. 2013; Wood et al. 2014).

### Effects on cognitive functions

Upper alpha based NF training had positive effects on memory functions in stroke patients, which is in line with previous findings in healthy people (Escolano et al. 2011, 2012, 2013, 2014; Angelakis et al. 2007; Nan et al. 2012). Subject A, who suffered from a bilateral SAH, showed the strongest deficits in memory functions prior to NF training. After NF training, his performance significantly increased in short- and long-term memory tasks compared to the pre-assessment. Before the start of the NF training, subject A’s performance in the Digit Span forward task and the VVM2 subscale *City map 2* was below average (T-scores < 40). During the post-assessment his performance in these two tasks was in a normal range. He also showed marginal performance improvements in working memory. Subject B, with lesions in the left hemisphere due to an ischemic stroke, also showed performance improvements in different memory tasks, although only the pre-post difference in the VVM2 subscale *City map 2* reached significance. Generally, upper alpha activity is associated with improved stimulus processing and inhibiting unnecessary or conflicting processes to the task being performed, which should foster the storage and retrieval of information and might explain the improvements in memory functions (Klimesch et al. 2007). A few prior single-case studies also found positive effects of NF training on cognitive functions in stroke patients (Rozelle and Budzynski 1995; Bearden et al. 2003; Putman 2002; Hofer et al. 2014; Cannon et al. 2010; Reichert et al. 2016). However, only a few studies explicitly investigated the effects of upper alpha based NF training on cognitive functions in stroke survivors (Doppelmayr et al. 2007; Kober et al. 2015b). Doppelmayr et al. (2007) reported the results of two NF training studies. In their first experiment, they found stronger positive effects of upper alpha based NF training on memory functions in a group of stroke patients compared to a control group of stroke patients that received relaxation training. In a second study, they could not replicate their findings. Upper alpha based NF training had comparable effects as theta based NF training and a control intervention (Doppelmayr et al. 2007). Nevertheless, in both experiments, they found an improved memory performance in stroke patients after upper alpha NF compared to the pre-test. The description of the stroke patients that participated in the study of Doppelmayer et al. (2007) is lacking, which diminishes the comparability of the present findings in stroke patients to the results of Doppelmayer et al. (2007). In line with the present results, Kober et al. (2015b) also reported on positive effects of upper alpha based NF training on memory functions. The present findings indicate that NF might be a suitable cognitive rehabilitation tool for stroke patients and should be investigated further in future studies. Research on NF for stroke rehabilitation is of special importance as positive effects of traditional cognitive trainings in this domain remain disputed (Nair and Lincoln 2007; Hoffmann et al. 2010).

Concerning the specificity of upper alpha based NF training, we found the strongest effects of UA NF on memory functions, except for subject B who also showed an improved performance in cognitive flexibility after NF compared to the pre-assessment. The majority of prior NF studies also linked upper alpha to memory functions, whereas performance improvements in other cognitive functions such as attention, inhibitory control, or cognitive flexibility were mainly found in theta/beta based NF training studies (Hofer et al. 2014; Kropotov 2009; Arns et al. 2009; Fox et al. 2005; Gruzelier 2014). In the NF literature, there are issues concerning specificity of NF training (Gruzelier 2014). To a large extent, our results indicate that there is specificity and independence regarding cognitive outcome such that performance enhancement in memory functions is specific to changes in UA frequency band while leaving other cognitive functions unchanged, except for cognitive flexibility.

Healthy controls, who showed no deficits in cognitive functions prior to the NF training, showed no significant changes in cognitive performance when comparing the pre- and post-assessment.

### Effects on EEG Activity During Rest

Upper alpha based NF training led to a topographical normalization in EEG resting delta and upper alpha power in subject B, who showed pathological deviations in EEG topography prior to NF training compared to healthy controls. The healthy control group showed a fronto-central maximum of delta power during the pre- as well as during the post-test. This is in line with prior findings in healthy people showing that delta activity is largest over fronto-central brain regions (Niedermeyer and Lopes da Silva 2005; Emek-Savaş et al. 2015; Schmiedt-Fehr and Basar-Eroglu 2011). Upper alpha power was largest over bilateral parieto-occipital electrode positions in the CG (Klimesch 1999). Subject B, who had lesions in the left hemisphere due to a stroke in the left ACM, showed an increased delta power over the healthy, right hemisphere compared to the affected, left hemisphere during the pre-test. Furthermore, before the start of the NF training upper alpha power was more pronounced over right than over left parieto–occipital brain regions in subject B. Single-case analysis revealed that this hemispheric asymmetry in delta and upper alpha power was significantly larger in subject B compared to healthy controls during the pre-assessment. There is evidence that an increased delta activity over the unaffected hemisphere is associated with poor recovery, a bad health status and even with earlier death (Finnigan et al. 2008; Sheorajpanday et al. 2011; Tecchio et al. 2007; Zappasodi et al. 2007; Niedermeyer 2005; Rossini et al. 2003). A higher alpha power over the unaffected compared to the affected hemisphere in stroke patients with unilateral lesions was also linked to poor clinical recovery (Giaquinto et al. 1994; Tecchio et al. 2006). Generally, an increased activity over the unaffected hemisphere in the chronic state after a stroke is associated with “maladaptive plasticity” (Rossini et al. 2003). An increased delta power over the unaffected hemisphere in subject B might be explained by a reduced blood flow in the unaffected hemisphere (Giaquinto et al. 1994) since there is evidence that delta power is negatively correlated with rCBF (Tolonen and Sulg 1981; Finnigan and van Putten 2013). In this context, studies in patients with infarctions of the ACM showed distant effects of some strokes such as reduced cerebral blood flow and reduced metabolic rates in contra-lesional regions, which might be related to the spill-over of EEG slowing into the healthy hemisphere (Niedermeyer 2005). Tecchio et al. (2007) also found increased delta power values in the unaffected hemisphere in patients with unilateral stroke in the ACM, which was related to a bad clinical long-term outcome prognosis. They also mentioned that a loss of excitatory input from the affected hemisphere might cause reduced metabolism in remote connected, healthy areas. Consequently, the authors concluded that clinical recovery might be paired with shifts of excitability balance between the affected and unaffected hemisphere (Tecchio et al. 2007). In line with this assumption, subject B showed a more bilateral distribution of delta power, with a fronto-central maximum after NF training, which was comparable to the topographical distribution of delta in the CG. Upper alpha power also showed a shift from a maximum over the unaffected healthy hemisphere during the pre-assessment to a more central, bilateral maximum during the post-assessment. The changes in topography of delta and upper alpha power after NF training in subject B compared to the pre-test might be a sign of “normalization” of neural activity. Single-case analysis revealed that the difference in EEG power between the left and right hemisphere in subject B regained values similar to healthy elderly controls after NF training. This reduction in pathological delta and upper alpha asymmetry in subject B was accompanied by cognitive improvements. The return toward homeostatic conditions of pathological brain mechanisms is in line with previous findings that showed that clinical recovery after stroke is accompanied by a reduction of inter-hemispheric asymmetries (Tecchio et al. 2006; Giaquinto et al. 1994; Rossini et al. 2003; Finnigan et al. 2006; de Vos et al. 2008). Rossini et al. (2003) also mentioned that an effective recovery after stroke is associated with a gradual normalization of the initially excessive intensity as well as with a normalization of the balance of hemispheric activation away from predominant activation in the unaffected, healthy hemisphere.

The observed topographical changes in delta and upper alpha power might be a sign of brain plasticity processes due to NF training in chronic stroke. Functional recovery after stroke is generally sustained by brain plasticity involving synaptogenesis, dendritic arborisation, as well as synaptic and axonal recruitment (Rossini et al. 2003; Sheorajpanday et al. 2011). Such brain plasticity processes might be fostered by NF training, since NF enables a direct access to neural activity, which might alter or accelerate functional reorganization in the brain following stroke (Nelson 2007). Our results indicate that NF can lead to brain plasticity processes in chronic stroke patients. According to Rossini et al. (2003), reorganization in response to therapeutic interventions can be studied best in patients in the chronic stage of stroke. Any recovery is likely to be the result of the specific intervention, since the probability of spontaneous recovery is negligible at this stage (Rossini et al. 2003). Furthermore, the probability that topographical changes in EEG power found in subject B between pre- and post-assessment were due to random fluctuations in the EEG signal is relatively low. Sheorajpanday et al. (2009) investigated the general reproducibility of EEG spectral power and asymmetry of EEG activity in stroke patients. They found relatively high Cronbach alpha values ranging from 0.95 to 0.99 for both EEG power and asymmetry indices (Sheorajpanday et al. 2009). These results show that EEG measures are robust parameters with a high reproducibility in stroke patients.

Beside changes in EEG topography, subject B showed an increased upper alpha power over parieto–occipital sites after NF training compared to the pre-test. The healthy controls also showed numerically increased upper alpha power values when comparing the pre- and post-resting measurements, however, these differences were not statistically significant. Single-case analysis revealed that subject B showed a significantly higher increase in upper alpha power after NF training compared to the pre-test than the CG. There is evidence that alpha power increases with clinical recovery in stroke victims (Giaquinto et al. 1994; Finnigan et al. 2007). An alpha amplitude attenuation is also generally indicative for cortical injury (Finnigan and van Putten 2013; Finnigan et al. 2007; Klimesch 1999). In healthy people, an increased tonic alpha power is associated with good cognitive performance, mainly memory performance (Klimesch 1999). Hence, the increase in upper alpha power due to NF training in subject B might be related to the improvement of cognitive functions.

Subject A, who suffered bilateral lesions due to a SAH, showed no hemispheric asymmetries in EEG power, neither before nor after NF training. This indicates that pathologic hemispheric asymmetries in EEG activity, which could only be observed in subject B with a left ACM stroke, might be specific for mono-hemispheric brain lesions. This is in line with prior assumptions that lateralization of slow activity such as delta power may be indicative of the primarily involved hemisphere (Niedermeyer 2005). Subject A also showed no prominent topographical alpha power maximum during rest. In this context, Niedermayer (2005) mentioned that the EEG of patients with SAH shows diffuse changes, disorganization, and a disruption of the posterior alpha rhythm, which might explain the missing topographical alpha power maximum over parieto–occipital sites in subject A.

Subject A showed cognitive improvements although no changes in EEG resting activity could be observed when comparing the pre- and post-assessment. Probably because of the diffuse and disorganized EEG and the disruption of the posterior alpha rhythm, which might be caused by the SAH, the method of EEG might have been not sensitive enough to disclose brain plasticity processes in subject A caused by NF (Niedermeyer 2005). Nevertheless, subject A could learn from NF training. He was able to voluntarily increase his upper alpha power within the training sessions and showed cognitive improvements.

We observed differences in EEG activity during resting conditions between stroke patients and controls only in the delta and upper alpha frequency range. For instance, subject B showed no hemispheric asymmetries in the theta, beta or gamma frequency range before the start of the NF training. Prior studies that showed deviant EEG activation patterns in stroke patients constantly reported abnormalities in the delta frequency range (Giaquinto et al. 1994; Finnigan et al. 2007, 2008; Finnigan and van Putten 2013; Fernandez-Bouzas et al. 2000; Claassen et al. 2004; Sheorajpanday et al. 2011; Tecchio et al. 2006). The majority of prior studies also demonstrated pathological alpha activity after stroke (Giaquinto et al. 1994; Finnigan et al. 2007; Finnigan and van Putten 2013; Sheorajpanday et al. 2011; Claassen et al. 2004; Tecchio et al. 2006; Fernandez-Bouzas et al. 2000). However, a few studies also reported on pathological theta or beta activity after stroke, which could not be observed in the present investigation (Giaquinto et al. 1994; Tecchio et al. 2006, 2007; Finnigan and van Putten 2013).

An important difference between prior studies and the present investigation is that in prior studies alpha power was defined within a broader frequency range of about 8–12 Hz (Giaquinto et al. 1994; Sheorajpanday et al. 2011; Claassen et al. 2004; Tecchio et al. 2006; Finnigan et al. 2007). In the present study, we split up the alpha frequency band in a lower (8–10 Hz) and upper (10–12 Hz) alpha power range, as suggested by Klimesch (1999). Upper and lower alpha power are linked to different cognitive processes. While lower alpha activity is associated with attentional processes that are relatively task- and stimulus-non-specific, upper alpha activity is specifically related to (semantic) long-term memory performance. Especially, search and retrieval processes in (semantic) long-term memory are reflected by upper alpha oscillations (Klimesch 1999). Furthermore, we chose upper alpha as feedback frequency during NF training, since prior NF training studies indicated positive effects of upper alpha based NF training on memory functions (Escolano et al. 2011, 2012, 2013, 2014; Angelakis et al. 2007; Nan et al. 2012). Hence, the increased resting state upper alpha power, which we observed in subject B and also marginally in the CG when comparing the multi-channel EEG measurements performed during pre- and post-assessment, might be indicative for specific improvements in memory functions due to upper alpha based NF, such as enhanced memory retrieval. In contrast, the absence of changes in lower alpha power might be a sign that upper alpha based NF had no effects on general attentional functions.

Changes in delta power due to NF training were comparable between the eyes-open and eyes-closed resting condition. In upper alpha power, topographical shifts between pre- and post-test could only be observed in the eyes-closed condition. Generally, alpha power is more pronounced when they eyes are closed than during eyes-open conditions. When participants open their eyes, EEG oscillations in the alpha band decrease in amplitude or disappear completely. This well-known and robust phenomenon in called “alpha blocking” (Niedermeyer and Lopes da Silva 2005; Kirschfeld 2005; Pfurtscheller and Lopes da Silva 1999). Probably, topographical shifts in upper alpha power could only be observed in subject B during the eyes-closed condition because alpha oscillations were more pronounced during this resting measurement compared to the eyes-open condition. Subject B also showed larger upper alpha amplitudes during the eyes-closed condition compared to subject A and the healthy CG. However, it is well known that alpha frequency shows large inter-individual differences (Klimesch 1999).

## Conclusions

Here we showed that upper alpha based NF training had (i) positive effects on memory functions and (ii) led to neuronal plasticity processes in chronic stroke victims. Initial pathological EEG activation patterns normalized after repeated NF training in a patient with unilateral stroke. Although changes in EEG activity during rest could only be observed in a stroke patient with unilateral stroke, both stroke patients could benefit from NF training. The NF training protocol was feasible for stroke patients with memory deficits and may represent a new rehabilitation strategy suitable to overcome some of the usual pitfalls of traditional cognitive rehabilitation. NF seems to be an alternative, innovative and easy-to-use cognitive rehabilitation tool since the electrical activity of the brain is affected directly and, therefore, the cortical substrates of cognitive functions themselves (Nelson 2007).
